# Heterogeneity in global gene expression profiles between biopsy specimens taken peri-surgically from primary ER-positive breast carcinomas

**DOI:** 10.1186/s13058-016-0696-2

**Published:** 2016-04-01

**Authors:** Elena López-Knowles, Qiong Gao, Maggie Chon U. Cheang, James Morden, Joel Parker, Lesley-Ann Martin, Isabel Pinhel, Fiona McNeill, Margaret Hills, Simone Detre, Maria Afentakis, Lila Zabaglo, Andrew Dodson, Anthony Skene, Chris Holcombe, John Robertson, Ian Smith, Judith M. Bliss, Mitch Dowsett, on behalf of the POETIC trialists

**Affiliations:** Royal Marsden Hospital, London, UK; Breast Cancer Now Research Centre, The Institute of Cancer Research, London, UK; Clinical Trials and Statistics Unit, The Institute of Cancer Research, London, UK; Lineberger Comprehensive Cancer Center and Department of Genetics, University of North Carolina, Chapel Hill, NC USA; Royal Bournemouth Hospital, Bournemouth, UK; Royal Liverpool University Hospital, Liverpool, UK; Faculty of Medicine & Health Sciences, Queen’s Medical Centre, Nottingham, UK; Present address: Kingston University, London, UK

**Keywords:** Breast cancer, Gene expression, Heterogeneity

## Abstract

**Background:**

Gene expression is widely used for the characterisation of breast cancers. Variability due to tissue heterogeneity or measurement error or systematic change due to peri-surgical procedures can affect measurements but is poorly documented. We studied the variability of global gene expression between core-cuts of primary ER+ breast cancers and the impact of delays to tissue stabilisation due to sample X-ray and of diagnostic core cutting.

**Methods:**

Twenty-six paired core-cuts were taken immediately after tumour excision and up to 90 minutes delay due to sample X-ray; 57 paired core-cuts were taken at diagnosis and 2 weeks later at surgical excision. Whole genome expression analysis was conducted on extracted RNA. Correlations and differences were assessed between the expression of individual genes, gene sets/signatures and intrinsic subtypes.

**Results:**

Twenty-three and 56 sample pairs, respectively, were suitable for analysis. The range of correlations for both sample sets were similar with the majority being >0.97 in both. Correlations between pairs for 18 commonly studied genes were also similar between the studies and mainly with Pearson correlation coefficients >0.6 except for a small number of genes, which had a narrow-dynamic range (e.g. *MKI67*, *SNAI2*). There was no systematic difference in intrinsic subtyping between the first and second sample of either set but there was c.15 % discordance between the subtype assignments between the pairs, mainly where the subtyping of individual samples was less certain. Increases in the expression of several stress/early-response genes (e.g. *FOS*, *FOSB*, *JUN*) were found in both studies and confirmed findings in earlier smaller studies. Increased expression of *IL6*, *IGFBP2* and *MYC* (by 17 %, 14 % and 44 %, respectively) occurred between the samples taken 2 weeks apart and again confirmed findings from an earlier study.

**Conclusions:**

There is generally good correlation in gene expression between pairs of core-cuts except where genes have a narrow dynamic range. Similar correlation coefficients to the average gene expression profiles of intrinsic subtype, particularly LumA and LumB, can lead to discordances between assigned subtypes. Substantial changes in expression of early-response genes occur within an hour after surgery and in *IL6, IGFB2* and *MYC* as a result of diagnostic core-cut biopsy.

**Trial registration:**

Trial number CRUK/07/015. Study start date September 2008.

**Electronic supplementary material:**

The online version of this article (doi:10.1186/s13058-016-0696-2) contains supplementary material, which is available to authorized users.

## Background

Molecular analyses of primary breast cancer for both research and patient management are now commonplace. Measurements may be made on diagnostic core-cut biopsies or surgical excisions that frequently comprise a very small fraction of the tumour. In so-called window-of-opportunity studies patients are exposed to medical therapy between diagnosis and surgery [1] and comparisons are made between samples taken at both time points. Valid interpretation of these studies depends on knowledge of any variability or systematic changes in the respective biomarkers that occur in the absence of treatment. Variability/heterogeneity may lead to false rejection of a true effect while systematic differences between diagnostic and surgical specimens may lead to artifactual changes being falsely ascribed to an intervention. For example, we have previously described the highly significant impact of specimen type (core-cut vs excision) on pAKT and pERK1/2 staining [2]. Pre-treatment/post-treatment comparison of biomarkers might also be affected by the taking of the diagnostic biopsy and changes due to cold ischaemia between resection and tissue stabilisation/fixation.

The effect of cold ischaemia time has been studied in small cohorts of breast cancer with up to 24 hours elapsed time before fixation, snap freezing or placement in RNA later [3–5]. No studies have directly examined the impact of the short time delay (20–60 minutes) resulting from sending specimens for X-ray, a frequent practice during breast cancer surgery to ensure the removal of the lesion (e.g. non-palpable mass, calcifications) and/or to check for adequate surgical margins, even in clinically palpable tumours. A small number of studies have evaluated gene expression changes over a longer period of time between biopsies [6–8]. For example, Jeselsohn identified 14 genes, including nine immune-related that differed between core-cuts and excision taken from 21 patients 6–65 days apart (mean 30 days).

Our primary objectives were to use genome-wide expression profiling to determine more comprehensively the variability and systematic changes in the expression of genes or pre-specified gene sets or subtype classifications (i) between two core biopsies taken (A) immediately after excision and (B) after sample X-ray and (ii) between diagnostic core biopsies (D) and surgical core biopsies (S) two weeks later in the absence of any intervention.

## Methods

### Patients and tissues

Study I. To answer the first objective we accessed tissues collated from a previously published study [2]. Core-cut biopsies (14-gauge needle) were taken from 26 surgical specimens and placed in RNAlater immediately after resection (sample A) and again after X-ray of the excised tumour (sample B). The time elapsed between samples A and B was recorded in the surgical report form.

Study II. To answer the second objective we accessed tissues from the no-treatment arm of The PeriOperative Endocrine Therapy - Individualising Care (POETIC) trial that randomized post-menopausal patients with primary ER+ breast cancer from 120 UK centres (2:1) to receive 2 weeks’ non-steroidal aromatase inhibitor (AI) or no-treatment for 2 weeks prior to surgery [1, 9].

At least one RNAlater-stored sample was available from 33.5 % (1493/4456) of patients or paired from 13.2 % (589/4456) of patients of the POETIC trial. A total of 227 control samples were subjected to RNA extraction. Expression analyses were conducted when a pair of RNA extracts was available with RIN >4. This amounted to 57 pairs of samples from control patients taken at diagnosis (D) and surgery (S).

### Ethics statement

Patient consent and ethics approval for the collection and analysis of breast cancer tissue samples was provided by the Royal Marsden Hospital for study I. Ethical approval for POETIC (Trial number CRUK/07/015) was provided by the National Research Ethics Service (NRES) Committee London – South East.

### Gene expression analysis, data pre-processing, data analyses and statistical methods

The detailed methodology is described in the Additional file 1.

In brief, extracted RNA was amplified, labeled and hybridized on Illumina global gene expression BeadChips (Illumina San Diego, CA, USA). Illumina raw data was extracted using GenomeStudio software and transformed, normalized and batch-corrected. Paired samples were excluded from further analysis if their fraction of detected genes was <30 % and probes were filtered out if they were not detected in any sample. Gene expression data from this study is deposited at GEO (http://www.ncbi.nlm.nih.gov/geo/query/acc.cgi?acc=GSE73237) with accession number GSE73237.

Entrez Gene ID was used as gene identifier in gene signatures. The HumanHT-12_V4_0_R2_15002873_B annotation file was used to map the Entrez Gene IDs to the corresponding Illumina probe IDs. Gene signature scores were weighted averages.

We evaluated three candidate gene sets: (i) metagene wound healing signature [10]; (ii) immune-response metagene [9]; and (iii) 13 of the 14 genes identified as changing in the Jeselsohn study [6] (SNAI1 was not detected on the Illumina platform). We also studied the effects on 18 pre-specified genes that we selected as being particularly relevant to breast cancer from prior studies.

Each tumour sample was classified into one of the five intrinsic subtypes based on the PAM50 classifier as described in the Additional file 1.

Pearson and Spearman correlations were used to assess the associations. Univariate paired or unpaired *t* tests together with multivariate permutation tests were used to identify differentially expressed genes between the paired samples. The significantly differentially expressed genes were subjected to Ingenuity Pathway Analysis (IPA). The significance of the difference between two correlation coefficients obtained in study I and study II respectively was calculated using the Fisher r-to-z transformation [11]. GraphPad Prism 6 (Graphpad Software Inc., La Jolla, CA, USA) was used for some of the statistical analyses in this study.

## Results

### Study I

Sufficient RNA was available from 26 sample pairs with up to 90 minutes between samples A and B. Three pairs were excluded due to low fraction of detected genes, leaving 23 pairs with a time interval of 20–60 minutes (median 30) for downstream data analysis. Patient demographics are described in Additional file 2: Table S1.

#### Variability in gene expression between samples

**Fig. 1:**
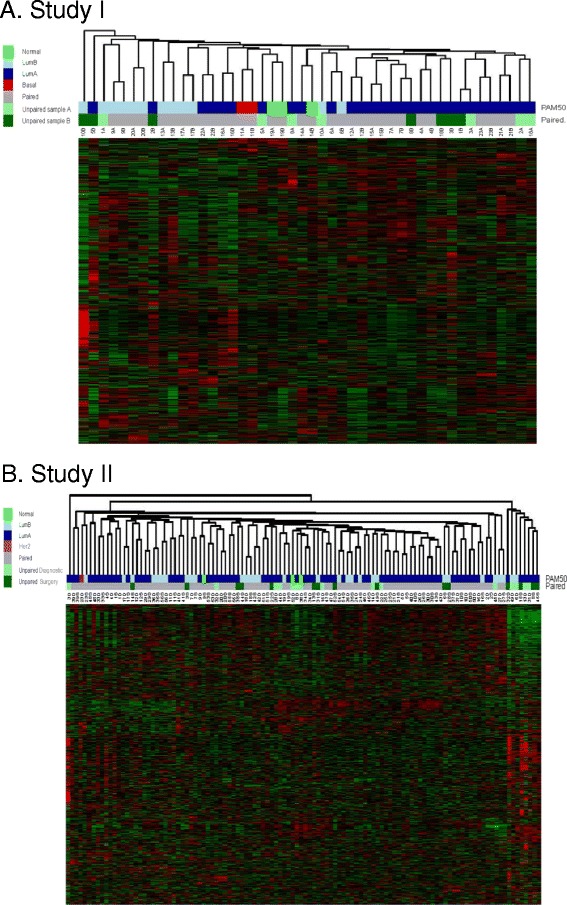
Hierarchical clustering with Euclidean distance and average linkage based on **a** study I: clustering of 24,395 probes and 23 pairs of samples; **b** study II: clustering of 32,332 probes and 56 pairs of samples. In brief, probes and samples were grouped based on similarities calculated using the Euclidean distance method and average linkage (Additional file 1: Supplementary information). Sample dendrogram bars were coloured according to PAM50 intrinsic subtypes and pairing of samples respectively. PAM50 color: *green* = normal; *dark blue* = LumA; *light blue* = LumB; *purple* = Her2-enriched; *red* = basal; *grey* = paired together: *light green* = unpaired first sample; *dark green* = unpaired second sample. *LumA* luminal A, *LumB* luminal B

On hierarchical clustering 16 (70 %) of the pairs clustered together (Fig. 1a). The correlation of the gene expression for the 24,395 probes between samples A and B provides an overall assessment of the similarity of transcriptional profiles between the samples. The Pearson correlation coefficient r values ranged from 0.91 to >0.99 (Additional file 3: Figure S1). Nine selected pairs in Additional file 3: Figure S2 represent the range of variability: three sets of three pairs with a coefficient >0.99, 0.98 or 0.91–0.94. Correlation was also determined between paired expression levels of 18 pre-selected genes frequently reported in breast cancer (Additional file 2: Table S2, Additional file 3: Figure S3). The correlation was above >0.6 and highly statistically significant for all genes, except for *MKI67* (r = 0.35, *p* = 0.10), *SNAI2* (r = 0.43, *p* = 0.04) and *PGR* (r = 0.52, *p* = 0.01) (Table 1). Upload of the full data set to GSE73237 (http://www.ncbi.nlm.nih.gov/geo/query/acc.cgi?acc=GSE73237) allows investigators to assess the correlation/variability of their genes of interest.

**Table 1:** Correlation of paired expression levels in five genes reported in breast cancer (complete list of 18 genes in Additional file 2: Table S2) and nine genes identified by Jeselsohn

		STUDY I					STUDY II				STUDY I vs. STUDY II	
		Gene symbol	R	*p* value	Geometric mean of B/A	95 % CI	R	*p* value	Geometric mean of S/D	95 % CI	Z value	*p* value (2 tail)
		BAG1	0.713	0.0001	0.971	0.946-0.996	0.734	<0.0001	1.043	0.984-1.106	-0.17	0.865
		MKi67	0.354	0.0978	1.009	0.962-1.058	0.522	<0.0001	0.977	0.930-1.027	-0.8	0.4237
		MAPT	0.847	<0.0001	0.806	0.692-0.938	0.811	<0.0001	1.108	0.965-1.273	0.44	0.6599
		PGR	0.522	0.0106	1.093	0.946-1.263	0.824	<0.0001	0.978	0.894-1.070	-2.25	0.0244
		SNAI2	0.430	0.0408	0.897	0.790-1.018	0.481	0.0002	0.940	0.838-1.054	-0.25	0.8026
Genes that significantly changed in Jeselsohn et al. (2013) [6]	(a) immune related	IGFBP2	0.583	0.0035	1.051	0.862-1.282	0.784	<0.0001	1.136	1.031-1.251	-1.48	0.1389
		IL6	0.712	0.0001	1.108	1.003-1.223	0.194	0.1525	1.167	1.079-1.262	2.65	0.008
		CD68	0.412	0.0509	1.065	0.889-1.272	0.464	0.0003	1.099	0.985-1.226	-0.25	0.8026
		CD14	0.553	0.0062	1.047	0.905-1.211	0.355	0.0074	1.017	0.901-1.148	0.96	0.3371
		CD52	0.755	<0.0001	1.085	0.923-1.276	0.436	0.0008	1.038	0.876-1.230	1.97	0.0488
		CD44	0.458	0.0278	0.927	0.788-1.091	0.816	<0.0001	0.952	0.890-1.019	-2.48	0.0131
		PPARG	0.315	0.1438	0.806	0.608-1.068	0.343	0.0096	0.993	0.870-1.132	-0.12	0.9045
		ADM	0.476	0.0217	0.931	0.720-1.204	0.544	<0.0001	1.122	0.964-1.306	-0.35	0.7263
		VEGFA	0.653	0.0007	1.043	0.967-1.124	0.647	<0.0001	0.991	0.930-1.055	0.04	0.9681
	(b) non-immune related	CENPF	0.781	<0.0001	1.039	0.913-1.183	0.729	<0.0001	1.062	0.959-1.176	0.46	0.6455
		MYC	0.509	0.0132	1.076	0.897-1.292	0.65	<0.0001	1.439	1.241-1.668	-0.82	0.4122
		CCNB1	0.413	0.0501	0.976	0.883-1.078	0.469	0.0003	1.010	0.919-1.107	-0.27	0.7872
		MAP1LC3B	0.598	0.0026	0.957	0.882-1.038	0.809	<0.0001	0.971	0.933-1.010	-1.65	0.099
		SNAI1	ND	ND	ND	ND	ND	ND	ND	ND	ND	ND

*ND* non-detected

#### Effect of time to fixation on gene expression

Using class comparison method with false discovery rate (FDR) <5 % no significant systematic differences in expression were found between samples A and B. However, 68 genes had a *p* <0.005 and fold change ≥1.25 (19 upregulated and 49 downregulated). Table 2 shows the top eight of these genes ordered according to fold change. The genes included early-response (*RGS1*, *RGS2*), mitochondrial ATP synthase (*ATP5C1*) and stress-response genes (*DUSP1*, *FOSB*). Ingenuity Pathway Analysis (IPA) of the 68 genes, using Benjamini-Hochberg multiple testing corrected B-H *p* value <0.05, identified six canonical pathways (Additional file 2: Table S3A). These were mainly associated with metabolism or signalling, the most significant being oxidative phosphorylation (B-H *p* value <0.005) and mitochondrial dysfunction (B-H *p* value <0.005). The top networks identified also included metabolism (Additional file 2: Table S3B).

**Table 2:** Top eight genes significantly different in paired samples of study I and study II

STUDY I					STUDY II				
Accession	Symbol	Parametric *p* value	FDR	FC	Accession	Symbol	Parametric *p* value	FDR	FC
NM_006732	FOSB	0.0014	0.138	2.08	NM_005252	FOS	<1e-07	<1e-07	4.00
NM_004417	DUSP1	0.0003	0.133	1.72	NM_002922	RGS1	<1e-07	<1e-07	3.23
NM_002923	RGS2	0.0003	0.133	1.59	NM_004417	DUSP1	<1e-07	<1e-07	3.13
NM_003407	ZFP36	0.0005	0.133	1.54	NM_000517	HBA2	<1e-05	0.003	-2.90
NM_033027	AXUD1	0.0001	0.087	1.49	NM_000518	HBB	<1e-05	0.006	-2.83
NM_004566	PFKFB3	0.0030	0.153	-1.48	NM_000517	HBA2	<1e-05	0.007	-2.64
NM_018955	UBB	0.0037	0.155	-1.46	NM_000558	HBA1	<1e-04	0.008	-2.39
NM_005063	SCD	0.0003	0.133	-1.45	NM_006732	FOSB	<1e-06	0.001	2.38

Change in expression of 116 genes correlated with time elapsed at *p* <0.005 (Additional file 2: Table S4) but none were significant by their adjusted *p* value. IPA of the 116 genes identified 28 pathways that were significantly changed at *p* <0.05. The most significant were adipogenesis and mitochondrial dysfunction and the main networks were inflammation and metabolic disease (Additional file 2: Tables S5 and S6). There were only two genes in common between the 68 (paired differences) and 116 (time-elapsed) gene lists (*SCD* and *AGPAT2* involved in fatty acid biosynthesis).

Two of the 18 genes pre-selected as frequently reported showed a modest but statistically significant difference between samples A and B: BAG1 (mean 3 % decrease, *p* = 0.026), MAPT (mean 19 % decrease, *p* = 0.007) (Table 1).

#### Analysis of candidate gene signatures and subtypes

There were no significant differences in the wound healing signature score [10] or an immune-response metagene [9]. One of the 13 genes identified to be changing in the Jeselsohn study (*IL6*) showed an 11 % increase (Wilcoxon matched-pairs signed rank test: *p* = 0.014) between samples A and B [6].

Concordance for intrinsic subtypes between the sample pairs is shown in Additional file 2: Table S7. The majority of these ER+ samples were luminal, as expected. Three tumours showed discordance between samples at time point A and time point B: two luminal A (LumA) samples at time point A were scored as luminal B (LumB) or normal at time point B; one LumB at time point A was rated as LumA at time point B. For each tumour, we calculated the numerical differences in the correlation coefficients to each of the LumA, LumB, and HER2-enriched centroids for each of samples A and B. As demonstrated in Additional file 3: Figure S4A, these three cases with discordant intrinsic subtypes between the time points A and B had the median values of numeric difference between their LumA and LumB centroid correlations of 0.08 and 0.32 when compared with a median difference of 0.54 (95 % CI 0.17–0.61) and 0.52 (95 % CI 0.10–0.54) for the concordant samples at time points A and B respectively.

### Study II

From the 57 pairs, 56 passed microarray quality control analysis. Patient demographics are described in Additional file 2: Table S1.

#### Variability in gene expression between samples

Seventy-three per cent (41/56) of pairs clustered together on hierarchical clustering (Fig. 1b). The correlation of the gene expression for the 32,332 probes between the two samples ranged from 0.86 to >0.99 with a median correlation of 0.97 (Additional file 3: Figure S5). As in study I, we evaluated the Pearson correlation coefficients between paired expression levels on 18 selected genes (Additional file 2: Table S2, Additional file 3: Figure S6). The correlation was above >0.6 except for *SNAI2* (r = 0.48), *MKI67* (r = 0.52), and *GPR160* (r = 0.55).

#### Gene expression comparison between baseline and surgery core

Thirty-nine genes (44 probes) were differentially expressed between biopsies D and S at FDR <5 % and fold change >1.25. The 39 genes included 11 early-response genes (*FOS*, *JUN*, *RGS1*), six stress-response/immune genes (*DUSP1*, *GADD45B*, *ATF3*), four snoRNA (*SNORD3C*, *SNORD3D*), four haemoglobin (*HBA2*, *HBB*) and five genes associated to breast cancer progression (*SIK1*, *TOB1*, *BHLHB2*). Table 2 shows the top eight genes identified. IPA analysis of the 39 genes identified 76 pathways affected (B-H *p* value <0.05) (Additional file 2: Table S8). Sixty per cent of the pathways identified were due solely to *FOS* and *JUN*. The most common enriched networks were proliferation and metabolism (Additional file 2: Table S9). None of the 18 pre-selected genes showed a statistically significant change between samples D and S (Table 1).

#### Analysis of candidate gene signatures and subtypes

There were no significant differences in the wound healing signature [10] or the immune-response gene signature [9] between samples D and S. Of the 14 detected significantly different genes described by Jeselsohn, two immune-related genes (*IL6* and *IGFPB2*) and one other gene (*MYC*) were significantly increased in their expression in sample S by 17 %, 14 %, and 44 %, respectively. The changes in *IL6*, *IGFBP2* and *MYC* did not significantly correlate with one another.

Most samples were luminal (Additional file 2: Table S7B). Six of 39 (15 %) tumours classified as LumA at baseline were classified as LumB at surgery, and four of 14 tumours classified as LumB at baseline were classified as LumA at surgery (29 %, 4/14). Among the 14 cases with discordant intrinsic subtypes between the baseline and surgery, the median values of numeric difference between their LumA and LumB centroid correlations were 0.089 (95 % CI 0.02–0.49) and 0.031 (95 % CI 0.12–0.34) when compared with median values of 0.50 (95 % CI 0.26–0.55) and 0.50 (95 % CI 0.26–0.53) for the concordant samples at baseline and surgery respectively (Additional file 3: Figure S4B). Interestingly, the one LumB/HER2-E subtype discordant case also had <0.3 between the LumB/HER2-E centroids.

### Study I and study II common genes

Nine of the top 20 genes significantly different with FDR <5 % and *p* <0.005 between samples D and S in study II were also significant with a *p* <0.05 between samples A and B in study I (Additional file 2: Table S10). These included *FOS*, *JUN* and other early-response genes.

**Fig. 2:**
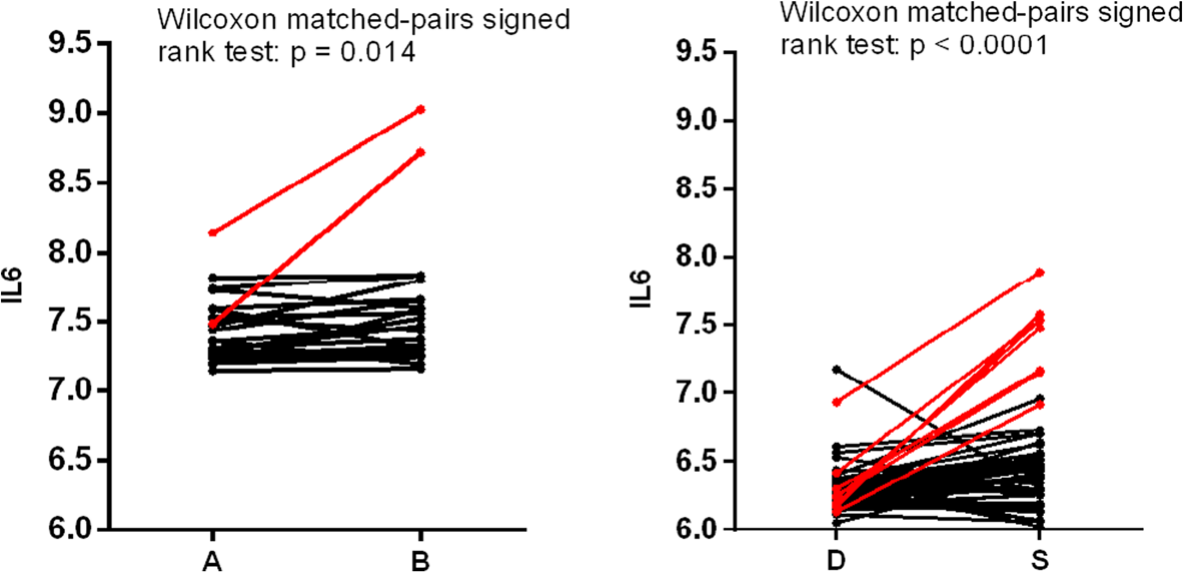
Line diagram of the paired *IL6* expression levels in study I and study II. Study I *IL6* expression levels of samples *A* and *B* and study II *IL6* expression levels at diagnosis (*D*) and surgery (*S*). Marked in *red* are samples with >50 % increase in expression

The changes in gene expression for *IL6* and *PGR* were significantly different between study I and II (Fisher’s r-to-z transformation, Table 1). *IL6* expression correlated positively between the two samples within study I but not in study II. This was due to the difference between the D and S samples varying substantially between tumours: there were large increases in *IL6* expression in a minority of samples while others remain largely unaffected (Fig. 2).

*PGR* expression was positively correlated between the paired samples in both studies. There was a significant tendency to an increase in study I (expression levels higher in time point B than A) and a decrease in study II (expression levels lower in time point S than D) that resulted in a marginally significant (*p* = 0.024) heterogeneity between the studies.

## Discussion

Multiple issues relating to intra-tumoural heterogeneity are at the forefront of contemporary molecular pathology. One concerns the degree to which a single core-cut biopsy can represent a biomarker’s expression across the tumour. We assessed this using a genome-wide approach. We also determined whether two common clinical practices around the time of surgery significantly affected the expression of particular genes or activation of certain pathways. Systematic changes resulting from either process would be relevant to any studies of excised breast cancer, since virtually all excisions occur after diagnostic core-cut and many will involve X-ray of the tumour before its fixation/stabilisation. Data from other studies may differ due to differences between the analytical platforms used.

The variability in whole genome expression data between tissue samples taken peri-surgically has been studied in only small tumour sets (greatest number 13, discussed below) [4–7]. Pure study of intra-tumoural heterogeneity is best conducted by taking multiple samples from a tumour at the same time. However, the systematic changes occurring in our studies were very modest and will have had little to no perceptible impact on the overall correlations observed. The range of correlations was similar across both studies and overall provided data on 79 tumours. The poorest of the correlations was 0.86 with the large majority being above 0.95 and several being >0.99. Thus gene expression overall shows only modest variability across tumours.

Most investigators are more interested in the variation in expression across the tumour for their gene or genes of interest. Our on-line data (http://www.ncbi.nlm.nih.gov/geo/query/acc.cgi?acc=GSE73237) will allow them to evaluate that. For illustration we chose 18 genes frequently studied in breast cancer. In general the correlation of the individual genes between the samples was higher for those genes with wide ranges, e.g. *TFF1* (6-log2 range) and *ERBB2* (5-log2 range) than those with narrow ranges, e.g. *SNAI2* (1.5-log2 range) and *MKI67* (<1.0-log2 range). The correlations between individual genes were all worse than those for the genome-wide analyses where there was an approximately 8-log2 range of expression.

We have previously reported that the 60-minute delay in fixation in study I had no significant impact on immunohistochemical expression of ER, PgR, Ki67, HER2, pAKT or pERK1/2 [2]. Similarly, no genes were found to differ at an FDR <0.05. However, several genes related to stress (e.g. *DUSP1*) and/or known as early-response genes (e.g. *RGS1*, *RGS2*, and *FOSB*) were among those most highly ranked according to change. In study II, where the larger number of samples provided greater statistical power, the same genes (e.g. *RGS1, FOSB* and *DUSP1*) or similar genes (e.g. *FOS*) ranked in the top ten genes with changed expression. This suggests that the changes in these early-response and stress pathways were true findings in both studies. It is important to note for study II that no record was made in POETIC of whether excised tumours were subject to X-ray before taking of RNAlater-stored core-cuts. At the Royal Marsden all impalpable tumours and most tumours resected via wide excision (totalling about 50 % of operations) are X-rayed. We have informally determined that similar approaches are in place across the UK. Some of the similarities in the genes changing between the studies may therefore have been due to a proportion of the tumours in study II being subjected to X-ray before stabilisation. It should be noted, however, that while the similarities in the gene changes between the two studies are consistent with delays due to X-ray being responsible in study II, there are multiple other factors that occur around surgery that could also contribute. These include the time taken for a sample to reach histopathology, where some centres may have taken cores for the POETIC study, and delays due to sentinel node biopsy, which may have occurred prior to the core being taken. Nonetheless the changes observed in study II are likely to represent those that occur between diagnostic and surgical samples in common practice and will affect the measurement/study of early-response genes in excised tumours.

Two smaller studies have assessed the impact of delay to fixation on global gene expression [4, 5]. In the Borgan study, changes in *FOSB* and *JUND,* while perceptible after 60 minutes, were much greater after 3 hours. The correlation of these changes with time since tumour removal make it likely that they are due to stress of tissue cutting and/or its exposure changed oxygen tension as opposed to the impact of other procedures around surgery such as anaesthesia. The pathway and network analyses undertaken with study I revealed changes in oxidative phosphorylation and mitochondrial dysfunction. This is also consistent with the exposure of the core-cuts to changed oxygen tension or ischaemia. The correlation of mitochondrial dysfunction also correlated quantitatively with time between core-cut taking and fixation supports this change being causatively associated.

Despite the lack of change in the pre-specified immune signatures *IL6* expression increased in both studies and was among the genes identified by Jeselsohn in a similar but smaller study. The change in *IL6* levels in study II was sufficiently heterogeneous between tumours to nullify the highly significant correlation between the A and B samples in study I, suggesting that the *IL6* changes were more related to the effects of the initial biopsy than to the short delays around surgery. *IL6* is a pleiotropic cytokine secreted by T cells and macrophages in both systemic and localised immune activation. Its role in breast cancer has been reviewed by Dethlefsen and colleagues [12]. Changes in *IGFBP2* and particularly *MYC* in study II also confirmed those seen in the Jeselsohn study, but there was little support for the other ten genes identified as significant in that study. Like *IL6* these two genes are widely studied in breast cancer. Interpretation of data on them must take account of the effects of diagnostic biopsies.

Some smaller genome-wide analyses between paired biopsies either side of surgery have been reported. Riis et al. [7] studied 13 patients with the time between diagnostic and surgical samples ranging between 2 and 8 weeks. As in the current study genes related to early response, including *FOSB* and to oxidative stress including *DUSP1* were differentially expressed between the two samples. Similar increases in early-response genes including *FOS* were also reported in 16 patients in whom fine-needle aspirates were taken presurgically and immediately after tumour excision but the time between samples was not stated [8]. Neither of these small studies, identified *IL6, IGFB2* or *MYC* as a changing gene but may have been due to their low statistical power.

There were no systematic differences in categorisation of the tumours into the intrinsic subgroups in either study but discordance was noted between the luminal A versus B subtypes, even after quality control of the RNA and removing technical platform bias with normalisation and standardisation of expression profiles. In study II, 15–20 % of tumours considered luminal A on one core-cut were typed as luminal B or normal-like on the other. Allocation of subtypes is made according to the highest correlation coefficient with the archetypical centroid for each subtype irrespective of the proximity of the correlations to the subtypes, although an early report [13] described 43/115 (37 %) of tumours as having a low correlation to any of the subtypes. Not surprisingly, we found that subtype discordances were largely associated with small differences between correlations with luminal A and luminal B centroids. The level of discordance in subtyping is important to appreciate given the prominence of intrinsic subtyping in clinical studies of breast cancer and its use for determining whether to allocate chemotherapy [14].

## Conclusions

These studies of both random and systematic variability of global gene expression in the context of presurgical study of breast cancer have revealed modest differences in most genes/pathways but confirmed substantial changes in the expression of early-response genes that appear to be due to ischaemia after surgery and in *IL6, IGFB2* and *MYC* that appear to be responses to initial core-cut biopsy. The data are relevant to all studies of breast cancer since excised tumours almost always have been preceded by core-cut. We provide a reference source (http://www.ncbi.nlm.nih.gov/geo/query/acc.cgi?acc=GSE73237) for others to assess the potential impact variability in the study of their own genes of interest.

## Additional files

Additional file 1: Supplementary information. Additional description of the materials and methods. (DOCX 24 kb) Additional file 2: Table S1. Demographics for study I and study II. **Table S2.** Correlation of paired expression levels in 13 genes reported in breast cancer (complementing Table 1). **Table S3.** A) Canonical pathways and B) top networks identified in study I. **Table S4.** Genes correlated with time elapsed in study I. **Table S5.** Top pathways identified from 116 genes correlated with time elapsed. **Table S6.** Top networks identified from 116 genes correlated with time elapsed. **Table S7.** Intrinsic subtype concordance between pairs. **Table S8.** Top pathways identified in study II. **Table S9.** Top networks identified in study II. **Table S10.** Top 20 genes identified in study I and their *p* value in study II. (XLSX 50 kb) Additional file 3: Figure S1. Paired correlations in study I. Correlation of detectable probes by Pearson correlation in 23 pairs of samples. **Figure S2.** Examples of paired correlations in study I. Correlation of detectable probes by Pearson correlation: the three samples with the highest correlations, median correlation and the lowest correlations. **Figure S3.** Correlation of 18 genes in study I. Pearson correlation of 18 genes commonly studied in breast cancer in 23 pairs of samples. **Figure S4.** Scatterplots of numeric differences between correlation coefficients to average gene expression profiles of intrinsic subtypes for each tumor in study I (S4A and B) and study II (S4C and D). Difference between luminal A and luminal B centroids (A and C), and luminal B and HER2-enriched centroids (C and D). *Open circle*: concordant subtype assignments between the two time points. *Triangle*: discordant subtype assignments between the two time points. **Figure S5.** Paired correlations in study II. Correlation of detectable probes by Pearson correlation in 56 pairs of samples. **Figure S6.** Correlations of 18 genes in study II. Pearson correlation of 18 genes commonly studied in breast cancer in 56 pairs of samples. (PDF 1479 kb)

## Abbreviations

AGPAT2: 1-acylglycerol-3-phosphate O-acyltransferase 2
AI: aromatase inhibitor
ATF3: activating transcription factor 3
ATP5C1: ATP synthase, H+ transporting, mitochondrial F1 complex, gamma polypeptide 1
AURKA: aurora kinase A
BAG1: BCL2-associated athanogene
BHLHB2: classic B basic helix-loop-helix protein 2
DUSP1: dual-specificity phosphatase 1
ER: oestrogen receptor
ERBB2: HER2, Erb-B2 receptor tyrosine kinase 2
FDR: false discovery rate
FOS: FBJ murine osteosarcoma viral oncogene homolog
FOSB: FBJ murine osteosarcoma viral oncogene homolog B
FOXA1: forkhead box A1
GADD45B: growth arrest and DNA-damage-inducible, beta
GPR160: G protein-coupled receptor 160
HBA2: hemoglobin, alpha 2
HBB: hemoglobin, beta
HER2-E: Erb-B2 receptor tyrosine kinase 2 enriched
IGFBP2: insulin-like growth factor binding protein 2
IL6: interleukin 6
IPA: Ingenuity Pathway Analysis
JUN: jun proto-oncogene
JUND: jun D proto-oncogene
LumA: luminal A
LumB: luminal B
MAPT: microtubule-associated protein tau
MKI67: marker of proliferation Ki67
MYC: v-myc avian myelocytomatosis viral oncogene homolog
pAKT: phospho v-akt murine thymoma viral oncogene homolog 1
pERK1/2: phospho extracellular signal-regulated kinase 1/2
PGR: progesterone receptor
POETIC: The PeriOperative Endocrine Therapy - Individualising Care
RGS1: regulator of G protein signaling 1
RGS2: regulator of G protein signaling 2
SCD: stearoyl-CoA desaturase
SIK1: sal-inducible kinase 1
SNAI2: Snail family zinc finger 2
SNORD3C: small nucleolar RNA, C/D box 3C
SNORD3D: small nucleolar RNA, C/D box 3D
TFF1: trefoil factor 1
TOB1: transducer of ERBB2, 1
TOP2A: topoisomerase (DNA) II alpha
