# Supplementary material for: Heterogeneity in global gene expression profiles between biopsy specimens taken peri-surgically from primary ER-positive breast carcinomas
Source: Breast Cancer Res. 2016 Apr 1;18:39. doi: 10.1186/s13058-016-0696-2 (PMC4818440; doi:10.1186/s13058-016-0696-2)
Supplement: Additional file 1: — Supplementary information. Additional description of the materials and methods. (DOCX 24 kb) [file 13058_2016_696_MOESM1_ESM.docx]

**Additional file 1. Supplementary Information**

*Gene expression analysis and data pre-processing*

Total RNA was extracted using miRNeasy (Qiagen, Sussex, UK). RNA quality was checked using an Agilent Bioanalyser (Santa Clara, CA, USA): samples with RNA integrity values of <4 were excluded from further analysis. RNA amplification, labelling and hybridization on HumanHT-12_V3 (study I samples) and HumanHT-12_V4 (study II samples) expression BeadChips (Illumina, San Diego, CA, USA) were performed according to the manufacturer's instructions. Illumina raw data was extracted using GenomeStudio software and was transformed and normalized using variance-stabilizing transformation, robust spline normalization method included in the R package (lumi) ([http://www.bioconductor.org](http://www.bioconductor.org/)). The data was then batch-corrected using the function (ComBat) in the R package (sva). Paired samples were excluded from further analysis if their fraction of detected genes was <30% and identified as outliers by a sample outlier detection function in the lumi package. Probes were filtered out if they were not detected in any of the samples (detection p>0.01). Gene expression data from this study is deposited at GEO (<http://www.ncbi.nlm.nih.gov/geo/query/acc.cgi?acc=GSE73237>) with accession number GSE73237.

*Gene Signatures*

Entrez Gene ID was used as gene identifier in gene signatures. The HumanHT-12_V4_0_R2_15002873_B annotation file was used to map the EntrezGeneIDs to the corresponding Illumina probe IDs. Gene signature scores were weighted averages as described previously [1].

We evaluated three candidate gene sets: i) metagene wound healing signature [2]; ii) immune response metagene [3] and iii) 13 of the 14 genes identified as changing in the Jeselsohn study [4] (SNAI1 was not detected on the Illumina platform). We also studied the effects on 18 pre-specified genes that we selected as being particularly relevant to breast cancer from prior studies.

Each tumour sample was classified into one of the five intrinsic subtypes based on the PAM50 classifier [5]. Prior to classification, technical bias between these data and the training data were minimized to ensure accurate calls across heterogeneous platforms. Under the assumption that The Cancer Genome Atlas ER+ cohort and the baseline specimens of the POETIC cohort were similar, gene-wise differences in the mean and variance of these two groups represent technical bias. These differences were removed from the POETIC and study I cohorts prior to PAM50 classification respectively.

Single sample intrinsic subtype prediction was performed by calculating a Spearman rank correlation coefficient between the 50-gene expression values of an individual sample compared to each of the average gene expression (centroid) values for Luminal A, Luminal B, HER2-Enriched, Basal-like, and Normal. The subtype classification for the study sample is assigned to the centroid with the highest correlation.

*Data analysis and Statistical Methods*

Pearson correlation coefficient was used to assess the association of the: i) detectable probes between the paired samples and ii) pre-selected genes between paired samples' expression levels. Univariate paired or unpaired T-tests together with multivariate permutation tests were used to identify differentially expressed genes between the paired samples. The significantly differentially expressed genes were subjected to Ingenuity Pathway Analysis (IPA). Pathways were considered as significantly altered if p<0.05 after using Benjamini-Hochberg Multiple Testing Correction. Wilcoxon matched-pairs signed rank test was used to evaluate the significance of the percentage increase of expression between pairs. The correlation of the difference in gene expression between biopsies and the length of the time interval between the biopsies was evaluated using Spearman rank correlation. The significance of the difference between 2 correlation coefficients obtained in study I and study II respectively was calculated using the Fisher r-to-z transformation [6] using the online calculator (<http://vassarstats.net/rdiff.html>). For each sample we calculated the following: (a) numeric difference of the LumA and LumB centroid correlation coefficients (i.e. LumA correlation coefficient minus Lum B correlation coefficient) and (b) numeric difference of their LumB and HER2-enriched correlation coefficients (i.e. LumB correlation coefficient minus HER2-enriched subtype correlation coefficient). Medians of these centroid correlation coefficients were reported and approximate 95% C.I. intervals were calculated using the adjusted bootstrap percentile method where appropriate [7]. No formal statistics comparison of the medians is performed.

GraphPad Prism 6 (Graphpad Software Inc.) was used for some of the statistical analyses in this study.

*Hierarchical clustering method*

To identify the clusters in gene expression between samples we used the Euclidean distance method and average linkage of sampleRelation function within the “lumi” R-package. The samples were then color annotated according to their PAM50 intrinsic subtypes (green = Normal; dark blue = LumA; light blue = LumB; purple = Her2-enriched; red = Basal) and whether or not the paired samples were clustered together (grey = Paired together: light green = Unpaired first sample; dark green = Unpaired second sample). In the final heatmaps of gene expression for the probes, they were generated based on the same clustering method (i.e. Euclidean distance method and average linkage), but keeping the order of samples. Function named colorRampPalette within R-package was used to specify the gradient of the colors.

**References**

1. Gao Q, Patani N, Dunbier AK, Ghazoui Z, Zvelebil M, Martin LA, Dowsett M: **Effect of aromatase inhibition on functional gene modules in estrogen receptor-positive breast cancer and their relationship with antiproliferative response**. *Clinical cancer research : an official journal of the American Association for Cancer Research* 2014, **20**(9):2485-2494.

2. Chang HY, Nuyten DS, Sneddon JB, Hastie T, Tibshirani R, Sorlie T, Dai H, He YD, van't Veer LJ, Bartelink H *et al*: **Robustness, scalability, and integration of a wound-response gene expression signature in predicting breast cancer survival**. *Proceedings of the National Academy of Sciences of the United States of America* 2005, **102**(10):3738-3743.

3. Dunbier AK, Ghazoui Z, Anderson H, Salter J, Nerurkar A, Osin P, A'Hern R, Miller WR, Smith IE, Dowsett M: **Molecular profiling of aromatase inhibitor-treated postmenopausal breast tumors identifies immune-related correlates of resistance**. *Clinical cancer research : an official journal of the American Association for Cancer Research* 2013, **19**(10):2775-2786.

4. Jeselsohn RM, Werner L, Regan MM, Fatima A, Gilmore L, Collins LC, Beck AH, Bailey ST, He HH, Buchwalter G *et al*: **Digital quantification of gene expression in sequential breast cancer biopsies reveals activation of an immune response**. *PloS one* 2013, **8**(5):e64225.

5. Parker JS, Mullins M, Cheang MC, Leung S, Voduc D, Vickery T, Davies S, Fauron C, He X, Hu Z *et al*: **Supervised risk predictor of breast cancer based on intrinsic subtypes**. *Journal of clinical oncology : official journal of the American Society of Clinical Oncology* 2009, **27**(8):1160-1167.

6. Fisher RA: **Frequency Distribution of the Values of the Correlation Coefficient in Samples from an Indefinitely Large Population**. *Biometrika* 1915, **10**(4):507-521.

7. Efron B: **Better Bootstrap Confidence Intervals**. *Journal of the American Statistical Association* 1987, **82**(397):171-185.
