# Supplementary figures and images for: Heterogeneity in global gene expression profiles between biopsy specimens taken peri-surgically from primary ER-positive breast carcinomas
Source: Breast Cancer Res. 2016 Apr 1;18:39. doi: 10.1186/s13058-016-0696-2 (PMC4818440; doi:10.1186/s13058-016-0696-2)

Figure S1

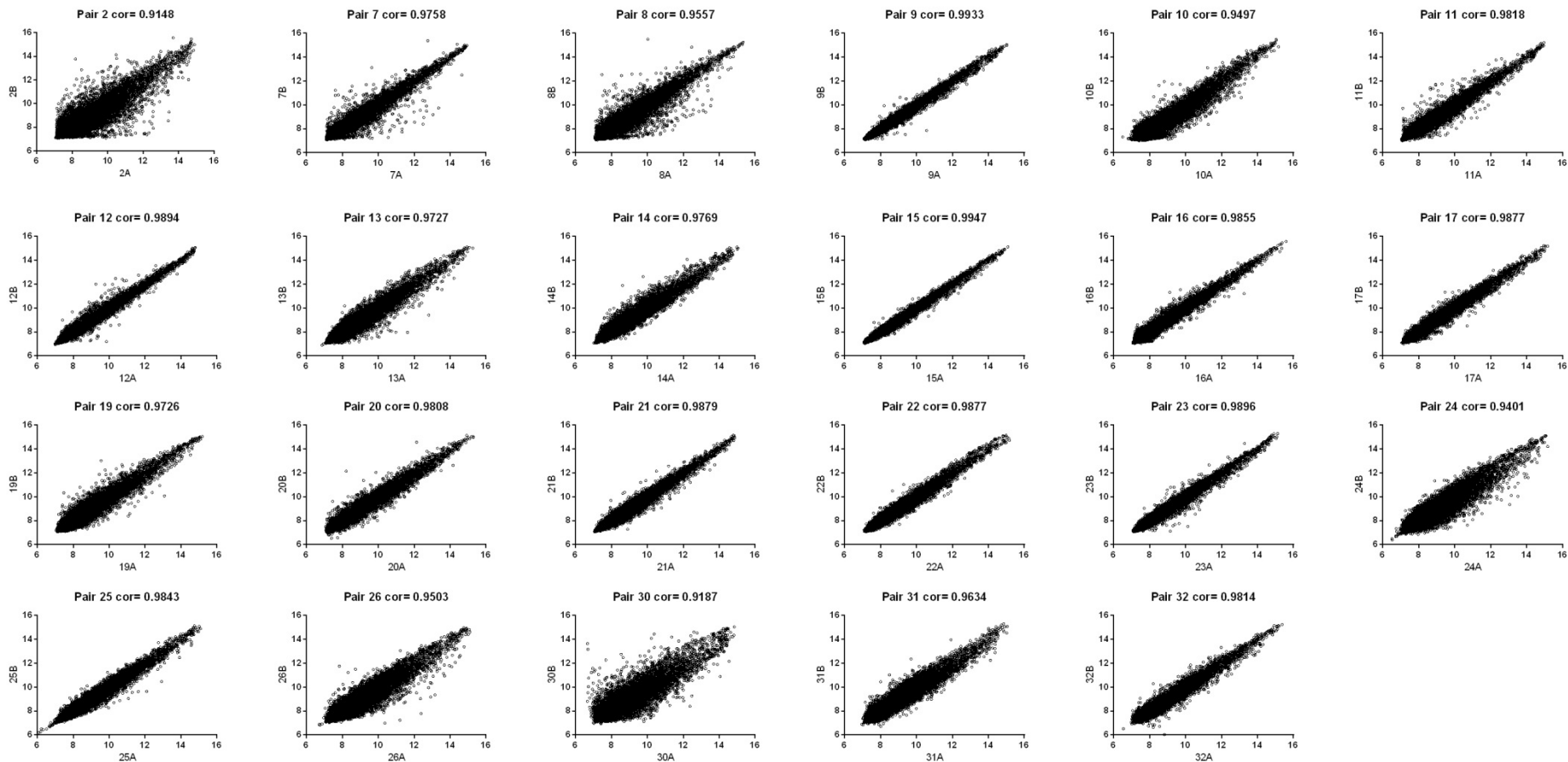

Figure S2

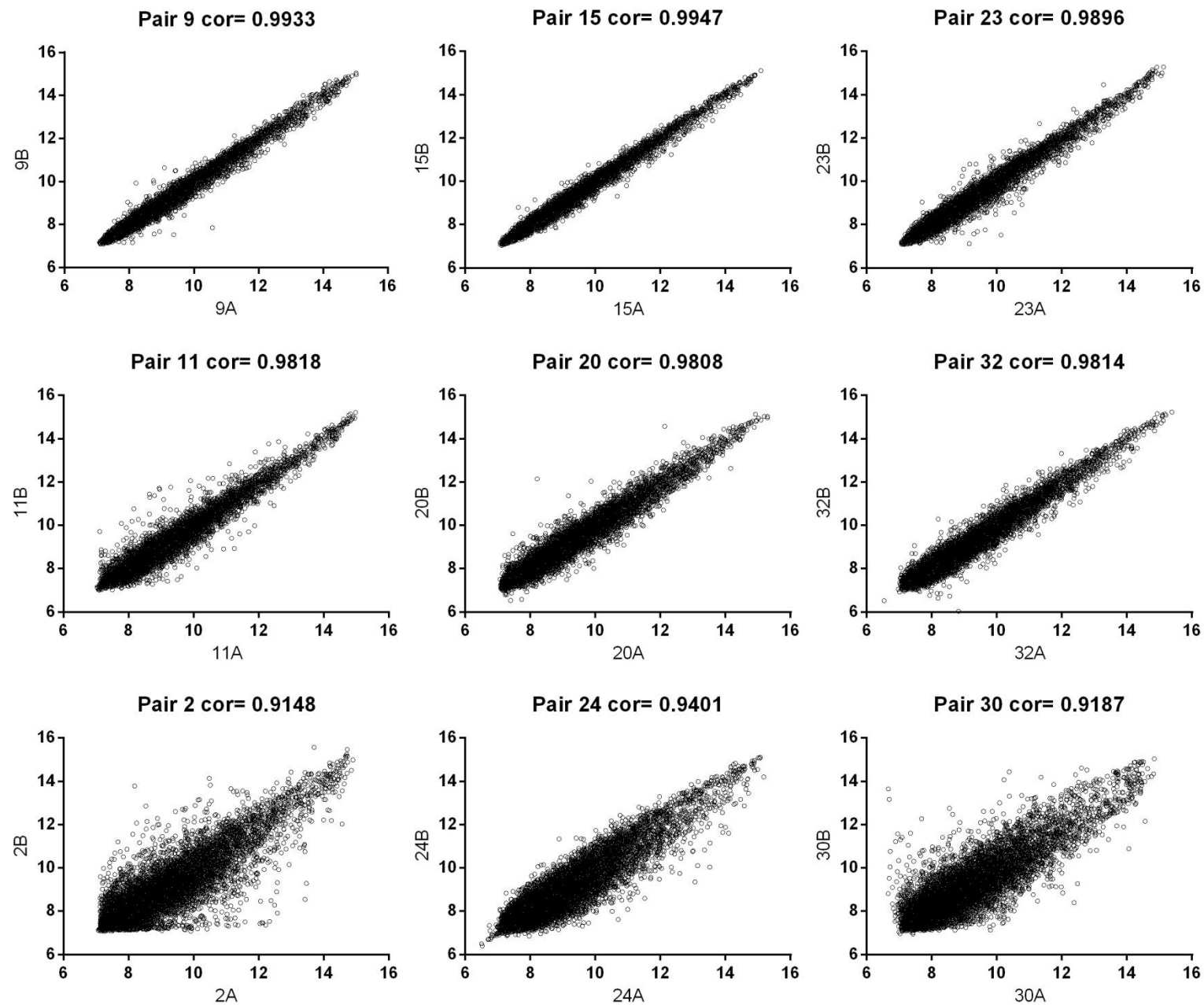

Figure S3

Study I

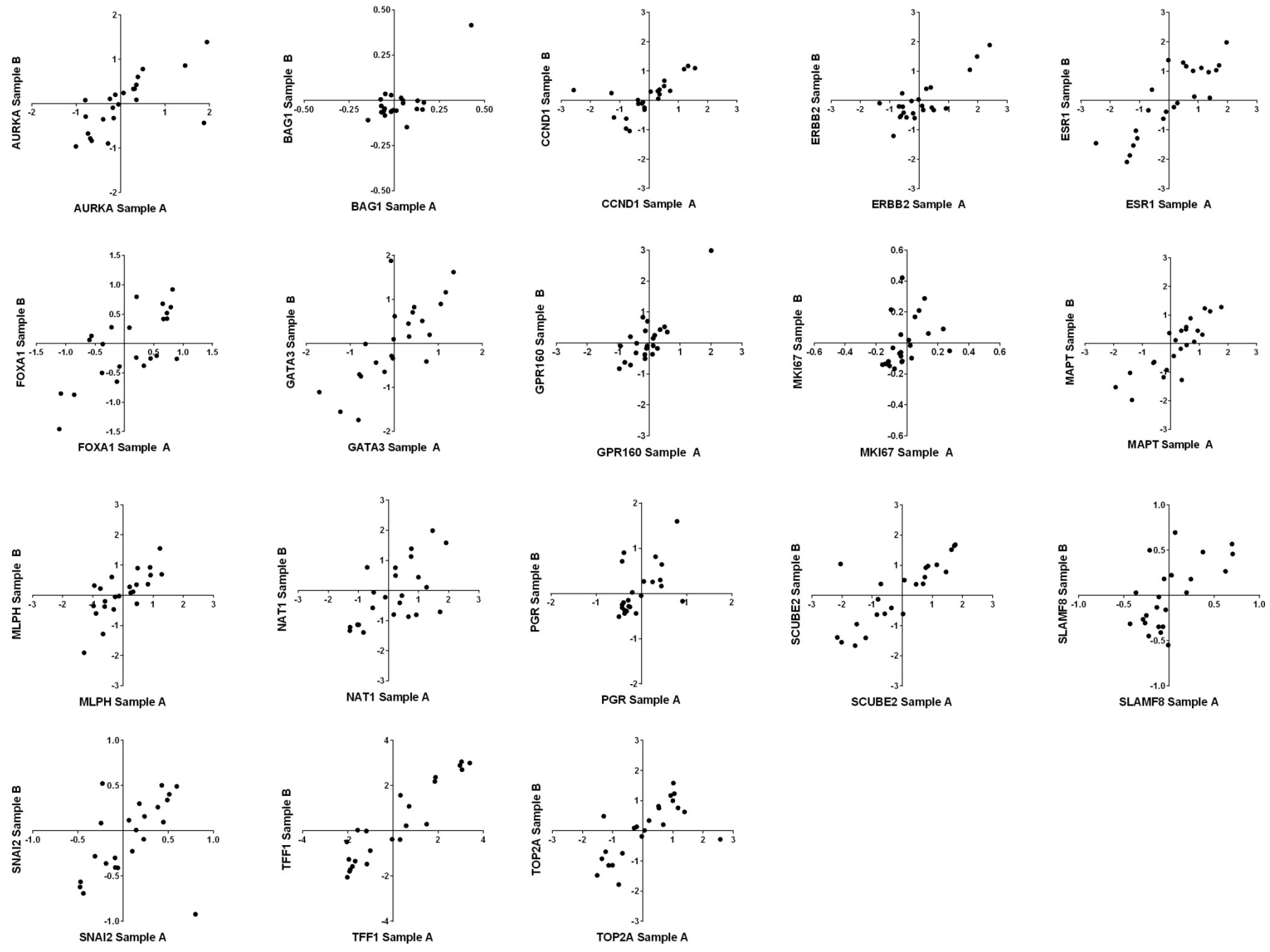

Figure S4

A. Study I

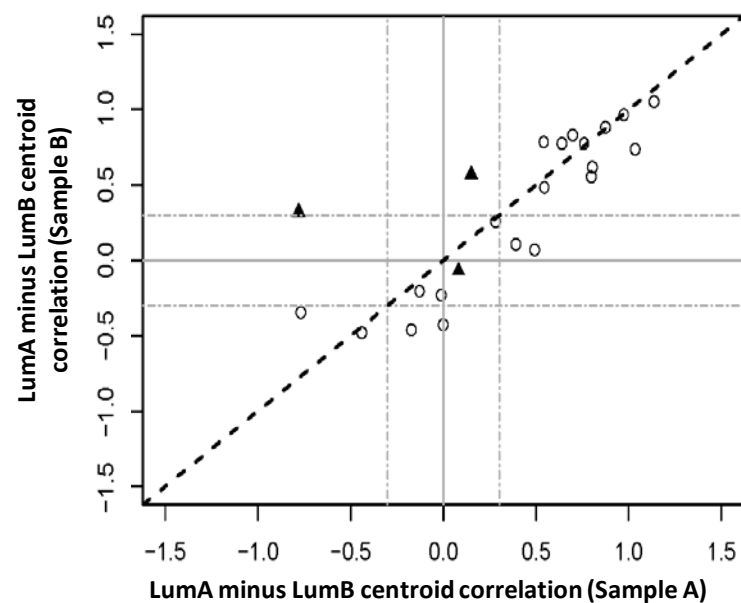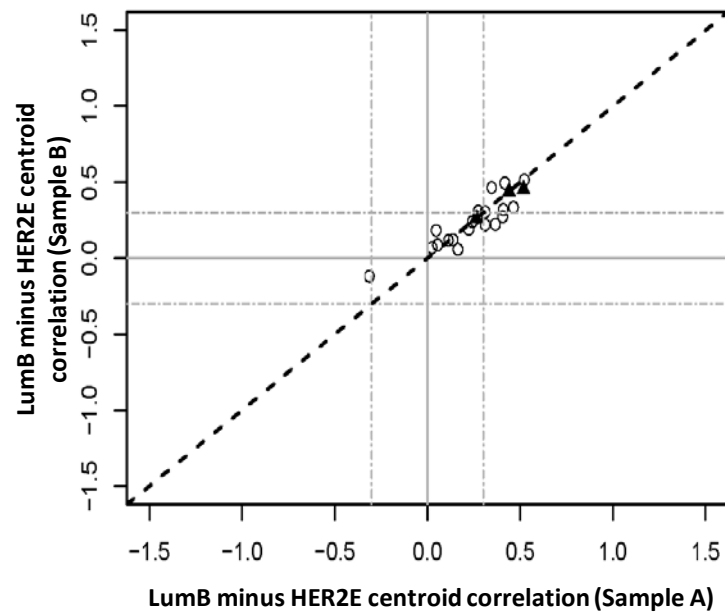

B. Study II

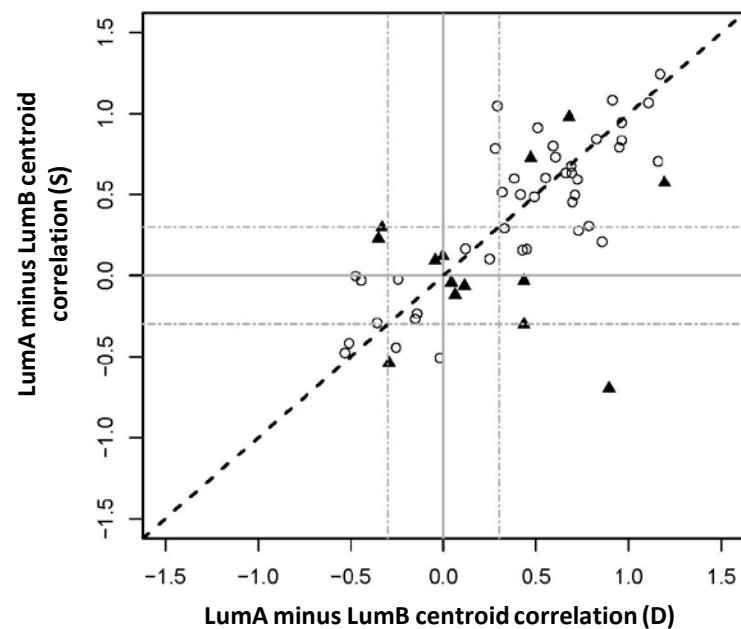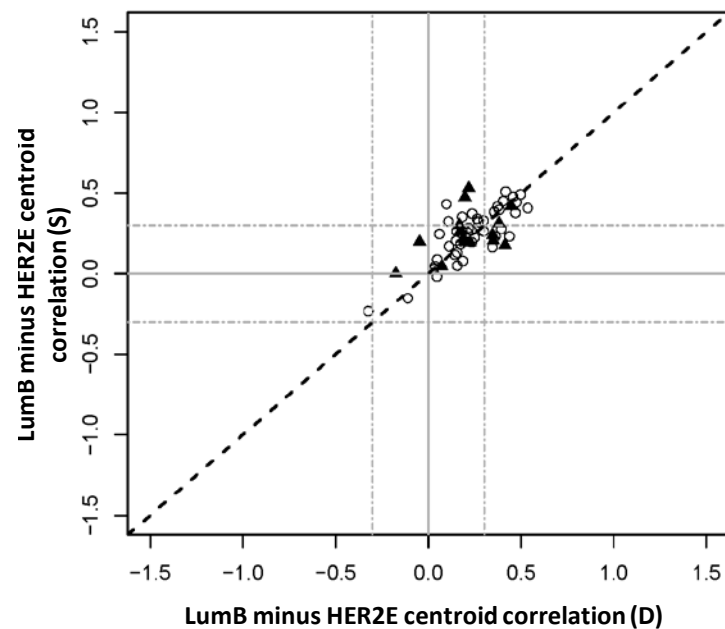

Figure S5

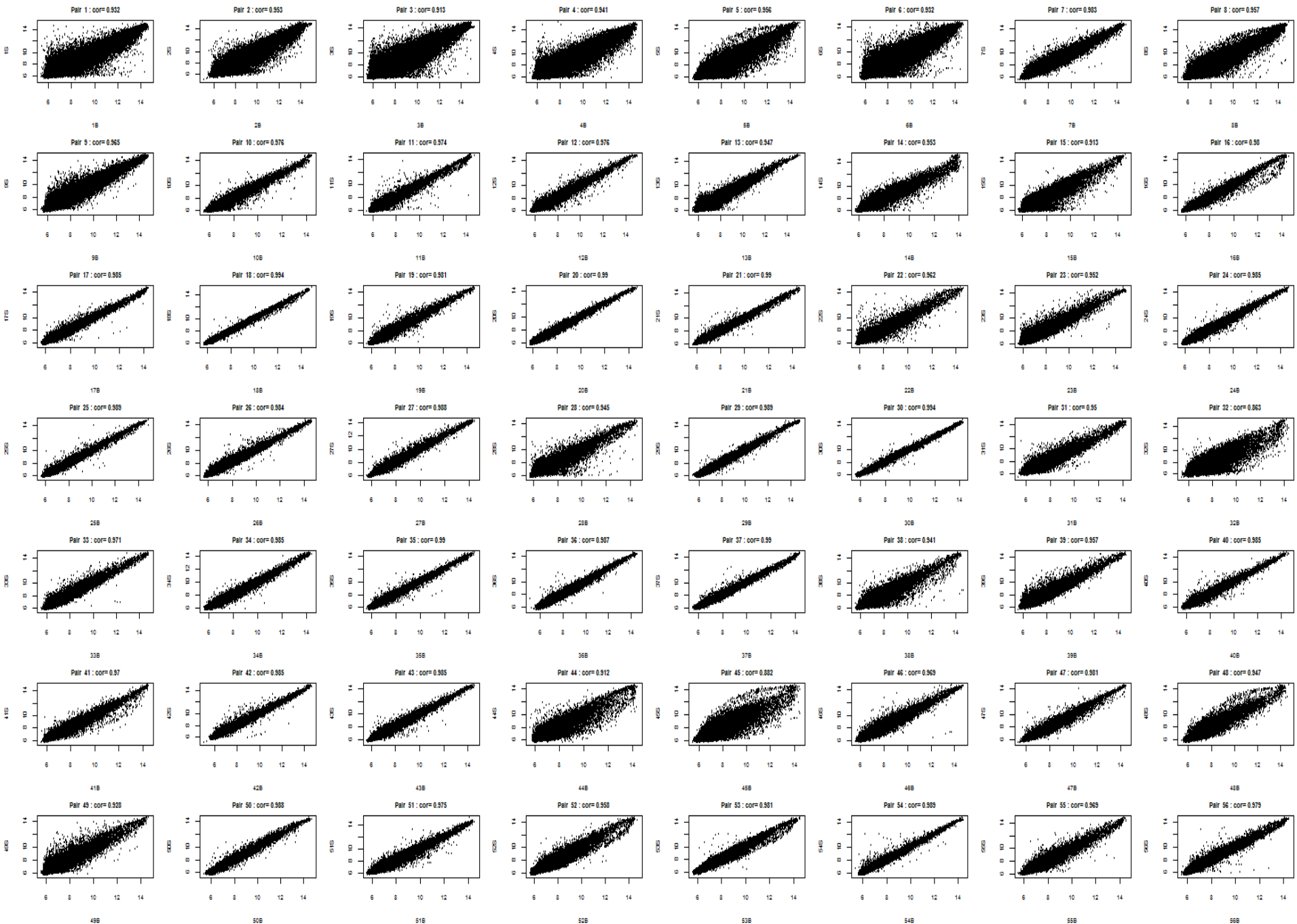

Figure S6

Study II

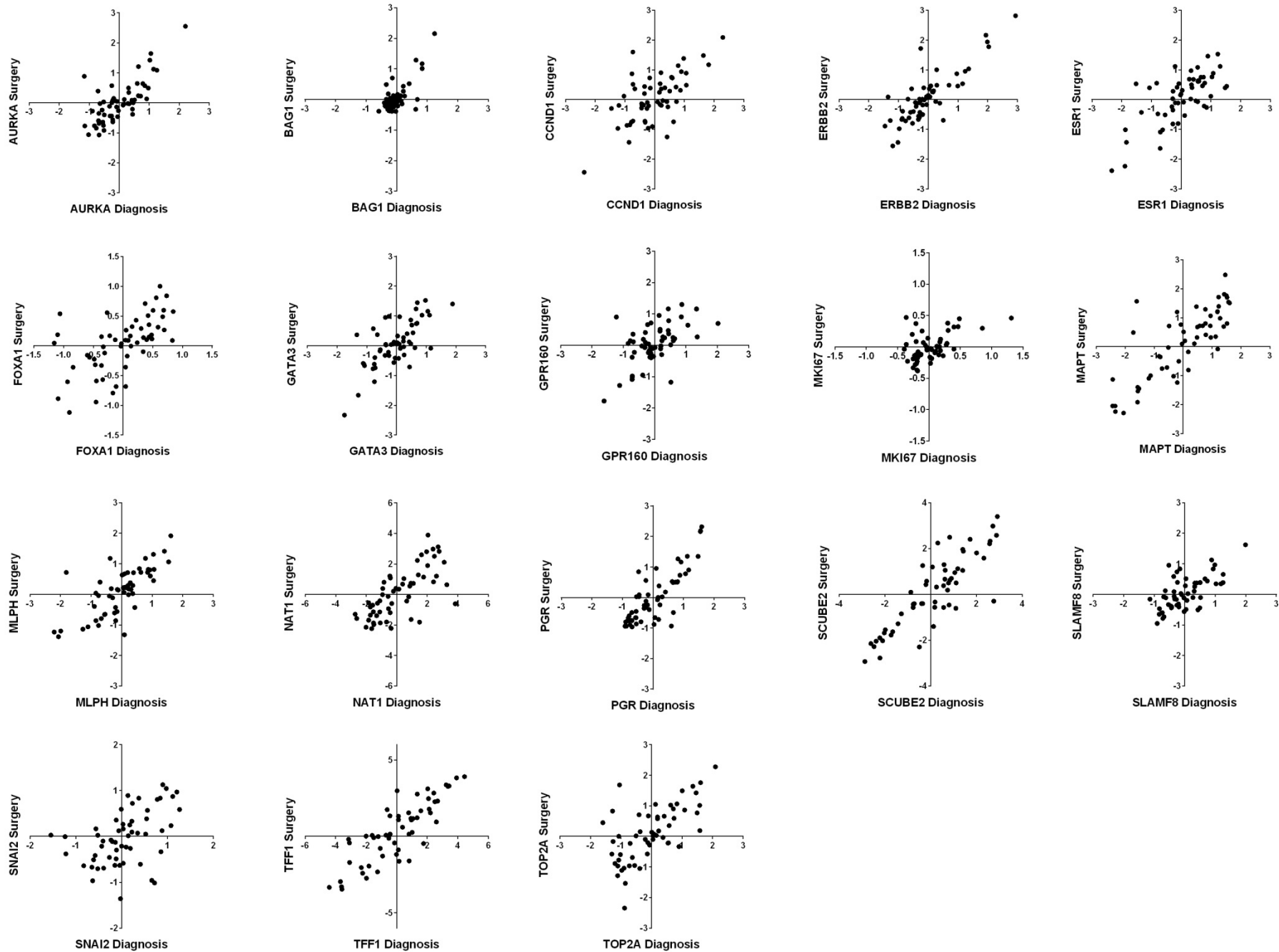

Supplement: Additional file 3: Figure S1. — Paired correlations in study I. Correlation of detectable probes by Pearson correlation in 23 pairs of samples. Figure S2. Examples of paired correlations in study I. Correlation of detectable probes by Pearson correlation: the three samples with the highest correlations, median correlation and the lowest correlations. Figure S3. Correlation of 18 genes in study I. Pearson correlation of 18 genes commonly studied in breast cancer in 23 pairs of samples. Figure S4. Scatterplots of numeric differences between correlation coefficients to average gene expression profiles of intrinsic subtypes for each tumor in study I (S4A and B) and study II (S4C and D). Difference between luminal A and luminal B centroids (A and C), and luminal B and HER2-enriched centroids (C and D). Open circle: concordant subtype assignments between the two time points. Triangle: discordant subtype assignments between the two time points. Figure S5. Paired correlations in study II. Correlation of detectable probes by Pearson correlation in 56 pairs of samples. Figure S6. Correlations of 18 genes in study II. Pearson correlation of 18 genes commonly studied in breast cancer in 56 pairs of samples. (PDF 1479 kb) [file 13058_2016_696_MOESM3_ESM.pdf]
